# Differences and commonalities in difficulties faced by clinical nursing educators and faculty in Japan: a qualitative cross-sectional study

**DOI:** 10.1186/1472-6955-11-21

**Published:** 2012-10-25

**Authors:** Maki Taniyama, Ichiro Kai, Miyako Takahashi

**Affiliations:** 1 Department of Nursing, International University of Health and Welfare, 1-2-25, Shiroyama, Odawara, Kanagawa, 2508588, Japan; 2 The University of Tokyo, 7-3-1 Hongo, Bunkyo-ku, Tokyo, 1130033, Japan; 3 Department of Public Health, Dokkyo Medical University, School of Medicine, 880 Kita-Kobayashi, Mibu, Tochigi, 3210293, Japan

## Abstract

**Background:**

To clarify the current state of communication between clinical nursing educators and nursing faculty members and the perceived difficulties encountered while teaching nursing students in clinical training in Japan.

**Methods:**

We collected data via focus group interviews with 14 clinical nursing educators, two nursing technical college teachers, and five university nursing faculty members. Interview transcripts were coded to express interview content as conclusions for each unit of meaning. Similar compiled content was categorized.

**Results:**

Difficulties in providing clinical training mentioned by both clinical educators and faculty members were classified into four categories: “difficulties with directly exchanging opinions,” “mismatch between school-required teaching content and clinical teaching content,” “difficulties with handling students who demonstrate a low level of readiness for training,” and “human and time limitations in teaching.” In some categories, the opinions of educators matched those of the faculty members, whereas in others, the problems differed according to position.

**Conclusions:**

The Japanese culture and working conditions may affect communication between clinical educators and faculty members; however, a direct “opinion exchange” between them is crucial for improving the clinical teaching environment in Japan.

## Background

Clinical practice is crucial in nursing to integrate theory and practice. Nursing students’ clinical knowledge, critical thinking for troubleshooting, and confidence in decision making can be augmented by applying the knowledge acquired in school during clinical practice [1,2]. Clinical practice is conducted in a variety of settings so that students may comprehensively and actively learn through nursing practice. University and school-affiliated faculty members, hospital-affiliated practice educators, clinical nursing educators, and staff nurses together teach students at the hospital. Rules and those who teach nursing to students in clinical practice differ depending on the country. In the United States, nursing students are trained primarily by faculty members [3], but staff nurses are also asked to teach students, leading to role-related problems between faculty members and staff nurses. In the United Kingdom, nursing constitutes a considerable portion of higher education, and staff nurses play an important role as mentors while teaching students [4]. As a mentor, a staff nurse facilitates learning and supervises as well as assesses students in the practice setting [4]. In Japan, the clinical educator plays an important role while teaching students in the clinical setting. A nurse with over 5 years of experience who has taken 240 hours of training sessions organized by the Japanese Nursing Association can become a clinical educator. The objective of the sessions is to gain an understanding of the meaning of clinical practice in nursing education, and to learn knowledge and skills for providing effective teaching in clinical practice. Clinical educators establish a human and material training environment so that educational encounters can be conducted through direct instruction on student-conducted patient care and conference-based instruction [5]. The clinical educator is affiliated with a ward of a hospital.

Generally, in Japan, students’ clinical experiences tend to be monitored by clinical educators with minimum involvement of faculty members. Faculty members belong to the University, give lectures on practice to nursing students in the school, and also supervise the nursing students in clinical practice.

As reported by Japan Academy of Nursing Education [6], approximately half of all nursing education institutions in Japan conduct clinical practice at hospitals established by the same organization, such as universities, governments and healthcare corporations. The other half of nursing education institutions which have no hospitals established by the same organization have to make the effort to secure hospitals which will accept their nursing students for clinical practice placements.

Some of the problems occurring in nursing student clinical practice are thought to be causally related to the teaching roles of persons in different positions, such as faculty members, clinical educators and staff nurses. A focus group interview (FGI) of nursing staff working in the acute care ward and of faculty members revealed the following: staff nurses preferred working with faculty members that work as nurses in the same ward; it is a burden to help students achieve their goals while handling a workload; staff nurses have insufficient information about the students’ capabilities and the faculty members have inadequate information-sharing capability [7]. In addition, the faculty members commented that they had insufficient time to develop the relationships necessary to accomplish the learning goals of the students; they had difficulty balancing research and clinical time; and they obtained insufficient patient information from staff nurses.

Several studies have shown that the “theory–practice gap” problem arises when educators from different positions are involved in clinical practice [8,9]. Employing educators to conduct integrated lectures and practical sessions has been suggested as a method to minimize this “gap” [9]. However, some studies [10-14] have noted several barriers to effective faculty member oversight of the clinical practice of students, most notably classroom priorities, workloads, meetings, time shortages, and the pressures of their own professional development [15]. In the United Kingdom, staff nurses are actively involved in nursing education to reduce the theory–practice gap. However, staff nurses are faced with ever-increasing pressures and responsibility, hindering their ability to provide optimal clinical teaching [16,17]. Several factors prevent staff nurses from competently performing this duty, including insufficient time, insufficient staffing levels, the need to prioritize patient care, and inadequate staff training [16-19].

In Japan, as previously explained, students’ clinical experiences tend to be monitored by clinical educators. Throughout their clinical experience, Japanese nursing students rarely, if ever, care for more than one patient at a time. As a result, they have little experience in organizing the management of complex tasks and responsibilities [3]. Results of a survey on nurse burnout in the United States, Canada, England, Scotland, Germany, and Japan [20,21] revealed that Japan had a high proportion of nurses aged below 30 years with high rates of burnout and low job satisfaction. This situation may be the result of nursing students in Japan undergoing training with inadequate involvement of school faculty members in busy wards predominantly staffed by young nurses.

Difficulties in clinical practice in Japan include differences in the perception and role of school and clinical training in journal discussions [22], investigations on problematic issues, the role of training, including forms of training not reflecting clinical conditions, a perception gap in educational content between faculty members and nurses, and inadequate cooperation among faculty members and nurses [22,23]. However, as few studies have compared the difficulties encountered by the clinical educators and the faculty members, it continues to be unclear whether the problems faced by nurses in Japan, the United States, and Europe are the same and whether differences of perception exist.

This study aims to clarify the perceived difficulties encountered while teaching nursing students in clinical training in Japan.

## Methods

### Design

A descriptive qualitative approach was chosen because we pursued presentation of the facts from the participants’ point of view and wanted to stay close to the data [24]. In this descriptive study, data were collected through FGIs with clinical educators, nursing technical college faculty members, and nursing-related university faculty members. Basch defined the FGI as a “Qualitative approach to learning about population subgroups with respect to conscious, semi-conscious, and sociological characteristics and processes” [25]. FGIs were selected because they enable the exchange of frank opinions between colleagues with similar roles and are effective in revealing inner difficulties related to teaching in clinical practice.

### Study participants

First, we planned to have FGIs at the affiliated community college of the researchers, so we made telephone calls to 13 hospitals, 9 universities and 13 nursing schools which are located one hour and half away from the community college. As a result, three hospitals, three universities, and one nursing school agreed to participate in the research.

Survey explanation letters were sent to the assenting institutions. Nursing directors at the hospitals, chair persons of nursing departments at the universities, and curriculum coordinators at the nursing school picked out appropriate clinical educators and faculty members according to the inclusion criteria.

### Clinical nursing educators

Inclusion criteria for clinical nursing educators specified that they had directly taught students basic nursing or adult nursing clinical practice within the past year.

Fourteen clinical educators from three hospitals participated in the FGIs. The average years of service as a nurse were 11.0 ± 5.0 years, with an average of 4.4 ± 3.4 years of teaching students. Most participants were graduates of technical college (N = 10), with two graduates from junior colleges, and two from universities. Analysis of affiliated schools of students receiving clinical practice indicated that the highest number of students belonged to technical colleges (N = 14), followed by universities (N = 5), and community colleges (N = 2).

### Faculty members

Inclusion criteria for faculty members specified that the candidates were required to have been in charge of basic nursing or adult nursing classes and have had directly instructed students within the past year. Seven faculty members from one technical college and four community colleges and universities participated in the FGIs. The faculty members’ average duration of service was 6.3 ± 6.3 years, with 9.1 ± 6.0 years’ experience as a nurse. Most faculty members graduated from community colleges (N =3), followed by three-year technical colleges (N =3), and university (N = 1). Degrees acquired included bachelor’s degree (N = 4) and master’s degree (N = 3). Four were assistant professors and one was an associate professor.

### Data collection

FGIs were conducted for 60–90 minutes with six groups comprising clinical nursing educators, nursing technical college faculty members, and nursing university faculty members in each group (Table 1). Interviews with faculty members were conducted in meeting rooms at the affiliated community college of the researchers, and interviews with clinical nursing educators were conducted in meeting rooms at the affiliated hospitals of clinical nursing educators. Six FGIs were conducted. The average duration of the interviews was 81 minutes. Although the university faculty member group included two faculty members from the same university, all other participants in the group belonged to different universities and were not acquainted. Questions asked in the interview included the following: “Describe in detail the problems of providing clinical practice,” “Talk about feelings of dissatisfaction with your present role as a faculty member/ clinical educator involved in training,” “What do you get personally by teaching nursing students?” and “What kind of environment or support might result in a more effective method of teaching in clinical practice?”. Within the interview guide, several issues that were brought up in one FGI were brought forward to subsequent FGIs. The interviews were recorded and transcribed with the consent of the interviewed participants.

**Table 1:** FGI groups and participants

**FGI group**	**Setting**	**Length of FGI**	**Participants**
Nursing Educator 1	A hospital	1:08:51	4 nursing educators
Nursing Educator 2	B hospital	1:29:53	3 nursing educators*
Nursing Educator 3	B hospital	1:18:53	3 nursing educators*
Nursing Educator 4	C hospital	1:22:43	4 nursing educators
Faculty members 1	Community college	1:22:13	5 faculty members (2 belonged to a community college, 3 belonged to universities)
Faculty members 2	Community college	1:22:24	2 faculty members (A technical college)

* We had two FGIs in B hospital, but participants were different between groups.

### Data analysis

The FGIs were analyzed focusing on the perceived difficulties encountered while teaching nursing students in clinical practice. As per Lofland and Lofland [26], interview transcripts were repeatedly read and coded to express interview content as conclusions for each unit of meaning. Similar compiled content was categorized. The data were coded by one person, who played roles as both coder and interviewer. The coding process was started after finishing all of the FGIs to reduce influences on the derivation of themes. Subcategories arose from the coding, and categories were extracted from the subcategories. Coding process were supervised by a researcher who is experienced in qualitative data analysis.

A member from each group was requested to check the validity of the analysis, but only two FGI participants joined in the member checking activity. Their opinions were solicited on the validity of the categories.

### Ethical considerations

Interviews were conducted after obtaining written consent and after explaining that survey collaboration would be independently undertaken and that uncomfortable questions were not required to be answered. Participants were assured that individuals and affiliated institutions would not be disclosed. This study was approved by the ethics committee of the Graduate School of Medicine, The University of Tokyo.

## Results

Difficulties in providing clinical practice teaching for nursing students mentioned by both clinical educators and faculty members were classified into four categories, as discussed below and as shown in Table 2. In some categories, the opinions of educators matched those of the faculty members, whereas in others, the problems differed according to position.

**Table 2:** Problems Perceived by Clinical Educators and Faculty during Clinical Training

**Categories**	**Sub categories**	
	**Clinical educators**	**Faculty members**
1. Difficulties with directly exchanging opinions between clinical educators and faculty members	1-1. Concerns about a worsening relationship with faculty	1-1. Concerns about a worsening relationship with educators
	1–2. Lack of confidence in teaching since educational background differs from students and faculty.	1–2 Difficulty of making critical comments because of having requested practice placement.
		1–3. Not complaining in the event of unsatisfactory teaching because of awareness of the burden on nurses
2. Mismatch between school-required teaching content and clinical teaching content	2-1. Teaching treatment-related techniques to students is difficult since the locus of responsibility is unclear.	2-1. Basic nursing techniques performed at clinical site differ from techniques performed in school
	2–2. Unaware of acquired learning/clinical practice goals.	2–2. Goals set for students differ between schools and clinical educators.
	2–3. Goals set for students differ between schools and clinical educators.	
3. Difficulties with dealing with students with low level of clinical training readiness	3-1. Difficulty of dealing with unwilling students	3-1. Difficulty of dealing with unwilling students
	3–2. Difficulty of dealing with emotionally immature students	3–2. Difficulty of dealing with emotionally immature students
	3–3. Difficulty in dealing with students for whom patient-centered thinking is difficult	3–3. Difficulty in dealing with students for whom patient-centered thinking is difficult
		3–4. Difficulty in dealing with students who may not be completely committed to nursing
4. Human and time limitations in teaching	4-1. Maintaining teaching continuity is impossible because educators change daily.	4-1. Maintaining teaching continuity is impossible because educators change daily.
	4–2. Time limitations and the number of students in the ward prevent educator from monitoring each students.	4–2. Burden of teaching role because of the responsibility of multiple wards and school lecture responsibilities
	4–3. Insufficient time for teaching and insufficient content for students by faculty.	

### Difficulties with direct opinion exchange between clinical educators and faculty members

Clinical educators and faculty members stated that the difficulty of directly exchanging opinions was the primary problem in clinical practice teaching for nursing students, which they avoided to escape being criticized or offending their counterparts.

(If wishing to directly convey negative opinions to the nurse) It is difficult to say something because I am afraid I will hear something like, “Faculty members would not come on request because they said they are busy, but they tell me to do it anyway.” (Faculty member)

(When the faculty member’s handling of an issue is questioned) I say something when I can, but sometimes I am afraid of hurting the faculty member’s feelings. I can talk with the faculty member when our relationship has been open and frank, but it’s difficult to talk with them when our relationship has not been good. (Clinical educator)

A clinical educator, aware of the importance of exchanging opinions for problem resolution, said that because of her experience, she gave up exchanging opinions with faculty members, and so she entrusts the problem solving to others.

(When a problem arose with a faculty member’s attitude) I spoke with the clinical instruction committee and a solution was obtained after the committee members spoke with the faculty member. From this incident, I determined that improving communication with the faculty members would benefit both sides. (Clinical educator)

Owing to the clinical educators’ “lack of confidence in teaching because their personal educational background differs from that of the students and faculty members,” we anticipated a tendency to avoid opinion exchange when faculty members and educators have different ideas on education. This category was extracted from the comments of educators and a faculty member who was experienced as a clinical educator.

(A case involving a difference in opinion with a faculty member) When our attitudes were similar, I could tell my opinion to a faculty member. But when our attitudes were not similar, I experienced difficulty in telling her my opinion. I tried to pretend to think that the faculty member’s perspective was better. (Clinical educator)

When I worked as a clinical educator, sometimes I was afraid of the nursing faculty. And I thought that the faculty might laugh if I said something incorrect. (Faculty member)

At the time of this study, approximately 80% of nurses were graduates of nursing school, with a few university graduates among the educators in charge of the education of students currently enrolled in nursing universities. Thus, clinical educators may tend to avoid direct opinion exchange because of the differences in educational background of faculty members and students. One faculty member spoke of an instance arising from consulting with an educator:

I have heard some educators say that teaching nursing university students can be difficult…the nursing university students seem to be more knowledgeable than the clinical educators. (Faculty member)

Conversely, some faculty members felt that they could not criticize training facilities and educators because they had requested the clinical practice opportunity. In general, the hospital has the authority to make the decisions about taking in the clinical practice program or not, so that faculty members refrain from commenting to avoid issues like clinical practice programs being refused or a decrease in student acceptance.

I heard a nurse manager say that they wanted to decrease the number of nursing students for clinical practice so that they would be able to admit more students from other schools. (Faculty member)

The faculty members tend towards “not complaining in the event of unsatisfactory teaching because of awareness of the burden on nurses” as faculty have past experience as nurses themselves; moreover, nursing burdens have increased owing to a changing medical environment with shorter periods of hospitalization. Although increasing clinical instructor educational influence on students is expected, expressing expectations of greater intervention are avoided because of the difficulty of such requests.

Nurses would say that sometimes they could not supervise the students when they were busy because of their responsibility to turn over the beds. And the tendency is especially strong when the hospitals face a new fiscal year when new nurses commence work. I can completely understand that. (Faculty member)

### Mismatch between school-required teaching content and clinical teaching content

Some faculty members are dissatisfied that clinical educators do not serve as role models for students because basic nursing techniques learned in school, including methods of taking vital signs and sanitary care, are not properly implemented at the clinical site: “Basic nursing techniques performed at the clinical site differ from techniques performed in school.”

With no strong opinions regarding their own basic nursing techniques, educators have stated that implementing the techniques expected by the schools at the actual clinical practice site is problematic: “Teaching treatment-related techniques to students is difficult since the locus of responsibility is unclear.” The Ministry of Health, Labour, and Welfare in Japan suggested that students should learn through clinical training the insertion of balloon catheters, administering shots, and endotracheal suctioning. But clinical educators think that it is difficult for nursing students to administer these treatments to patients at the clinical practice site [27].

We often entrust care-related teaching to the nurses. I was surprised when I attended a bed bath, for example, that the patient’s body may not often be covered with a towel or that the hot water may get lukewarm. If the individual nurses show consideration in such aspects towards patients, the students will also remember to consider such points. (Faculty member)

I know that the range of techniques that nursing students can perform has increased. One day, I asked a faculty member if a nursing student could perform a certain technique such as urinary catheter insertion. Her answer was “Patient care is the nurse’s responsibility, so the students may do it if the nurse allows.” This shows that the decision is up to the clinical educator. Therefore, we have to take responsibility if a problem arises, and thus we are hesitant to let the students perform certain techniques. (Clinical educator)

From the clinical educator’s view, a subcategory “Unaware of acquired learning/ clinical practice goals” was extracted. Before the clinical practice program begins, the faculty members and clinical educators have a meeting to discuss and confirm in writing and through verbal conversation which clinical practice goals, content, and techniques are to be taught. Despite this, educators do not always seem to completely understand these contents.

Although I received written materials on techniques to be taught and demonstrated in clinical practice at the meeting, I did not actually have the time to read them. (Clinical educator)

Even if the student expresses a desire to attempt many kinds of nursing techniques, it can be difficult because I am unsure of the extent of their training. (Clinical educator)

The faculty members probably did not completely inform the clinical educators about the students’ skills and about what they had learned in the classroom, because of the length of the meeting held before the start of the clinical practice program. Also, when clinical educators ask students if they have learned a particular technique, they often give an ambiguous answer. Additionally, there is not sufficient time to talk with clinical educators and faculty members during the clinical practice program. Therefore, clinical educators tend to avoid clarifying what the students have learned and to simply let the student perform various nursing techniques.

From the opinions of both groups, this subcategory was derived: “Goals set for students differ between schools and clinical educators.” The faculty members set a goal for students of providing the proper approach to care learned at school. This is based on a holistic assessment of the patient through the nursing process. However, at the clinical site, nurses tend to pay attention to the disease and its treatment rather than to the holistic point of view.

Because novice nurses should concretely understand pathophysiology and treatment of disease in the course of their work, their training emphasizes this point. Faculty members fear that clinical educators may instruct nursing students as they instruct novice nurses. Some educators have acknowledged that the anticipated achievements of nursing students may be confused with those of novice nurses.

Clinical educators do not understand that students already have a lot of homework; therefore, clinical educators give the students much more additional homework such as reviewing drug effectiveness for each drug the patients take and the results of blood tests. (Faculty member)

We expect students to ask many questions of us, but I have realized that we have this expectation because this is our approach to the novice nurses, to encourage them to ask many questions. However, students and novice nurses are not the same, and treating them the same would be stressful for the students and for us. Moreover, the staff nurses are probably unaware of this difference. (Clinical educator)

### Difficulties with handling students who demonstrate a low level of readiness for clinical practice

From the FGIs of both groups, the subcategories of “Difficulty of dealing with unwilling students”, “Difficulty of dealing with emotionally immature students” and “Difficulty in dealing with students for whom patient-centered thinking is difficult” were extracted. We categorize these subcategories as “Difficulties with handling students who demonstrate a low level of readiness for clinical practice.”

On dealing with unwilling students, clinical educators hoped that students “would become more willing,” while the faculty members could not comprehend the reasons for such a trait in students and have no idea of the immediate methods for improvement.

I want students to actively approach their training. Instead of expecting us to instruct them, we want them to approach us with their own goals for what they want to do. (Clinical educator)

Many students seem to wait for someone to approach them because they have been brought up in an environment where people offer help before the students have to ask for it. (Faculty member)

Although both faculty and educators noted “the difficulty of dealing with emotionally immature students,” these comments were more pronounced in the faculty members. Issues raised by faculty members included students blaming others without reflecting on themselves and not being good at coping with stress.

A student got angry at having to repeat the clinical practice for a certain thing. The student yelled, “What was wrong with the way I did it?” at the faculty member. (Faculty member)

Clinical educators have indicated that students are unable to completely control their emotions.

I do not know whether they are psychologically fragile, but some students cry easily; they start crying although I don’t get strict and am not angry. (Clinical educator)

“Difficulties of dealing with students for whom patient-centered thinking is difficult” was mentioned by both faculty members and clinical educators. Some students faced this problem in clinical practice, despite the emphasis of direct interaction with patients and on determining patient needs by observation. Problems mentioned included an unsuitable clinical environment, inability to obtain information from patients, and inability to incorporate the information obtained from patients into care. One clinical educator questioned whether sufficient time was being spent with patients.

Some students are insensitive to the opinions and ideas of patients and make decisions based on their own perspectives even after speaking with patients. Those students do not factor the patient’s circumstances into their nursing care plan, which is, therefore, invalid. (Clinical educator)

From the views of some faculty members, the subcategory of “difficulties of dealing with students who may not be completely committed to nursing” was extracted. This is usually recognized by faculty members before the clinical practice program, becoming clear from interviews between student and teacher at school and conversations with students of these schools. The tendency is more marked with students who chose nursing because of the encouragement of their parents or significant others. Faculty members have addressed the issues that such students are unable to devote themselves to clinical practice and that relationships between patients and the nursing process do not progress past a superficial level because of the absence of a clear goal to become a nurse.

Since a mother had urged her daughter to become involved in the medical field and she had qualified on the exam, the student enrolled in nursing school although she was not particularly interested. (Faculty member)

Few differences were observed between faculty members and clinical educators in in dealing with students who demonstrate a low level of readiness for clinical practice, as both groups struggled in a similar manner with these problem students.

### Human and time limitations in teaching

From the opinions of both groups, the category “human and time limitations in teaching” emerged. Concerns were raised that learning effectiveness in clinical practice is diminished because of human and time limitations in teaching.

Clinical educators and faculty members agreed that it is difficult for the same clinical educators to teach consistently during the clinical practice period and that thus “maintaining teaching continuity is impossible because educators change each day.” Clinical educators believed that time limitations from their simultaneous teaching and handling of other work duties were also detrimental to teaching continuity. Furthermore, “determining the state of each student is difficult because of the number of students training in the ward for clinical practice and because of time limitations.” One educator spoke of the problem in determining the direction of teaching because of an inadequate estimation of the learning level and thinking ability of the students.

Sometimes we are unaware of the progress of our students, their nature, or their ambitions because we are teaching and managing patients simultaneously. (Clinical educator)

Teaching five or six students in one ward is difficult. (Clinical educator)

In some facilities, one faculty member is in charge of multiple wards; therefore, they cannot be in one ward for very long, making it difficult to find the time to talk with students. Clinical educator dissatisfaction with faculty member limitations was particularly pronounced when teaching content for students was insufficient.

Because nurses face difficulties in supervising students who are poor learners, students should seek educational advice from faculty members. Often, even when faculty members come to supervise, it is brief. Therefore, we have had problems because faculty members were unavailable when students had difficulties in their studies. (Clinical educator)

Although the clinical nursing educators and staff nurses usually teach students when they provide direct care to patients, support from faculty members is expected when clinical educators and staff nurses are busy. This was a source of dissatisfaction.

Faculty members tended to limit themselves to just supervising students’ records and entrusted everything else to the clinical educators when they were in our ward and this was really difficult for us. (Clinical educator)

A faculty member who had been in charge of three wards spoke of factors in the “burden of the teaching role because of the responsibility of multiple wards and school lecture responsibilities,” including the difficulties of managing the wards and students, exchanging opinions with educators, and adjusting clinical practice content while being in charge of multiple wards.

## Discussion

Based on FGIs of clinical nursing educators and faculty members providing clinical practice programs in Japanese hospitals, the problems of teaching in clinical practice were classified into four categories, as discussed below.

### Difficulties with direct opinion exchange

The results indicated reluctant and insufficient opinion exchange by clinical educators and faculty members. This may aggravate the sense of alienation between the two groups. The awareness for the need of cooperation between the clinical and educational sector is widespread in Japan [22,23,28], and approximately 90% of nursing schools conduct meetings to explain clinical practice to clinical educators as an effort to improve cooperation with training facilities [29]. Nevertheless, there seems to be inadequate communication between clinical educators and faculty members at the teaching sites. One study has revealed that there have been difficulties in the UK in strengthening the relationship between the clinical and educational sectors due to insufficient time [10]. Although the faculty members in this study spoke of “not complaining in the event of unsatisfactory teaching out of awareness of the burden on nurses,” this awareness may make it difficult to request time to talk.

Concerning the clinical educators’ idea that they “lacked confidence in their teaching since their personal educational background differs from that of the students and faculty members,” Andrews and Roberts [30] in the United Kingdom found that nurses acting as mentors in clinical practice believe, “Many practitioners do not feel confident to teach and assess students at a different academic level to themselves.” This is a common problem that may be resolved by increasing awareness of major changes in curricula.

We found “concerns of a worsening relationship between faculty members and clinical educators” and “the difficulty of making critical comments because of having requested clinical practice opportunities” as reasons for insufficient direct opinion exchange, whereas studies from Europe and the United States have not mentioned these reasons. “Concerns of a worsening relationship between faculty members and clinical educators” may arise because the respondents were Japanese, who try to preserve harmony with others by avoiding direct criticism. Japan is considered a high context society where one takes another’s situation into consideration rather than engaging in direct verbal communication [31]. We observed this cultural characteristic in the relationships of clinical educators and faculty members, which lacked frank opinion exchange and involved vague information exchange.

“The difficulty of making critical comments because of being in the position of having requested the clinical training opportunity” was a category extracted solely from faculty members. Securing new training hospitals has proven to be problematic for some communities in Japan as the establishment of new nursing universities continues. Because admission for training is typically at the discretion of the hospitals, faculty may refrain from making comments that would lead to training being refused because of faculty members’ behavior. Accepting clinical training programs for the hospitals provides the opportunity to evaluate nursing quality by improving nurse teaching abilities. Creating a system whereby hospitals benefit from offering clinical training guidance is important for conducting effective training.

### Mismatch between school-required teaching content and clinical teaching content

The lack of direct opinion exchange between clinical educators and faculty members may influence the issue of “mismatch between school-required teaching content and clinical teaching content.” The three-part articles by nursing community college faculty members and nursing chiefs introduced 20 years ago in Japan indicated that clinicians were unaware of the differences in goals for each training progression and of the importance of nursing progress records to increase thinking power and decision-making ability. In addition, the articles reported the inability of faculty members to propose specific learning methods for each training progression [22].

For faculty members, the goal for students in providing nursing care is to demonstrate a profound understanding of the physical and psychological aspects of patients while properly administering basic nursing techniques learned in school, such as sanitary care and physical assessment. However, in the clinical setting, basic nursing techniques that are given high priority in school are neglected.

Clinical educators observe that teaching treatment-related techniques is difficult because it is unclear who will be responsible for the techniques that students must administer during training, including medication, injections, and urethral catheterization. Allowing students to administer techniques after confirming their technical level places an enormous burden on clinical educators and staff nurses. Langan [7] observed this tendency in the United States.

Gaberson and Oermann [32] emphasize that academic skill and clinical ability are required for nursing faculty; however, Japanese faculty members who engage in faculty practice are uncommon [29]. Efforts to improve faculty members’ clinical skills are not specifically supported in Japanese nursing education. Faculty members’ clinical skills may be improved and communication with the teaching hospitals may be facilitated, as in Langan’s study, by engaging faculty members in clinical practice and in applying theory to actual patient care [7]. Students can be more efficiently guided and education content learned in school can be utilized to teach correct methods at the site if faculty members also administer treatment-related techniques to patients.

Clinical educators and faculty members believed that “goals set for students differ between the schools and the clinical educators.” Both groups were concerned that students and new nurses were being taught in the same manner. The clinical educators’ educational role in clinical practice should be one of “questioning and prompting investigation” to support the development of students’ ability of thinking rather than the “teach knowledge, show techniques” method used in training new nurses [33]. Faculty members need to become role models for education and to verbalize the aim of each educational relationship to make clinical educators aware of these differences.

### Dealing with students who demonstrate a low level of readiness for training

Although the proportion of students with a low level of readiness for training was not determined in this study, all clinical educators and faculty members in FGIs commented on the difficulties of relating with such students.

Unwilling students with low motivation may negatively influence other students and increase the time that faculty members and clinical educators spend on their issues, thereby reducing teaching time with other students, exhausting the faculty members and clinical educators, and rendering them unable to make a fair evaluation [34,35].

Unwillingness, emotional immaturity, and lack of patient-centered thinking can be signs of psychological problems, such as students having family problems or depression, low sense of self-esteem, and so on. Some problems might be difficult to solve during clinical practice programs. Faculty members and clinical educators should determine whether the problem might clear up or not, and shouldn’t hesitate to refer students who might have psychological problems to counselors or psychiatrists who are not directly related to their education.

For issues regarding “difficulties of dealing with students not completely committed to nursing” anticipated before clinical training, a complete understanding of the thoughts and feelings of the student is necessary, offering opportunities for introspection by the student on whether they truly want to become a nurse and supporting the student in exploring changing career paths.

### Human and time limitations to teaching

In Japan, faculty members and clinical educators often work together in teaching nursing to students in clinical practice. However, clinical educators must handle student education when faculty members cannot teach at the clinical site.

Twenty percent of the clinical educators who participated in these FGIs were completely devoted to teaching; the other 80% taught while handling a slightly lower patient load than usual.

The assessment of each student’s learning context and achievement by clinical educators is difficult because so many students are assigned to one ward. These findings are consistent with those of Langan [7] and Clifford [10].

Although clinical educators can allocate time to support the care provided by students, it is difficult for them to supervise students when not performing direct care. It is also difficult for clinical educators to check each student’s nursing process and learning context.

When clinical educators are too busy, they expect faculty member to teach students direct care and check their assessment, but faculty members are often away from the clinical site. Allocating time for discussions with faculty members is not possible. Thus, a sense of inadequacy in teaching and uncertainty about the school and faculty members may arise.

Because of manpower constraints, some faculty members are in charge of multiple wards. They still feel the strain of their teaching role, whereas clinical educators consider the involvement of the faculty members to be inadequate. Gaberson et al. stated, “Quality of teaching may be problematic for faculty members who predominantly teach in the classroom or change practice settings frequently.” [32] The phenomenon of faculty members teaching in a specific training ward, with some latitude in time, is required to maintain education quality.

Some clinical educators have expressed dissatisfaction that faculty members entrust the supervision of patient care provided by students to clinical educators. For faculty members, clinical ability diminishes as teaching experience increases, leading to the possibility that faculty members may lose confidence in their ability to conduct patient care. Furthermore, a conflict of roles may easily arise since nursing faculty members function as educators and nurses [36]. Although as faculty members they want to prioritize support for student learning over patients, as nurse-educators, they want to prioritize the fulfillment of patient needs. As Hewison and Wildman [37] stated, “The clinical area is geared up for practice rather than education,” and Cahill [38] found that “Teaching and learning activities among nurse, educator, and student occur only when the work had been completed.” Faculty members must understand that these feelings are prevalent. However, students are the main priority of those involved in nursing education. The result of proper training of the students will be passed on to the patients as excellent care. Faculty members and clinical educators must ensure that excellent care is the ultimate goal of nursing education.

### Implications

From the FGIs, most of the subcategories are similar between the groups of faculty members and clinical educators. However, some sub categories were different between the groups. For example, in the category of “Difficulties with direct opinion exchange between clinical educators and faculty members”, there was “Not complaining in the event of unsatisfactory teaching because of awareness of the burden on nurses” as a subcategory derived from faculty members. If clinical educators recognize that some faculty members think that way, it can be a cue to start discussions to solve the problems arising in the clinical practice programs. Rather than criticizing clinical educators, this shows acceptance of the difficult situation of nursing educators. Faculty members should not think “I can’t ask the clinical educator because they are too busy”, or “I should wait until they perceive a need of the students.” Faculty members must ask clinical educators to figure out a way to meet the student’s needs even in difficult situations. Fear of criticism might be a cause of lack of communication between faculty members and clinical educators. To build mutually supportive relationship between both groups, others should be encouraged with positive feedback.

Lack of communication between faculty members and clinical educators affects the category, “Mismatch between school-required teaching content and clinical teaching content”. For instance, if “Goals set for students differ between schools and clinical educators”, faculty members and clinical teachers should discuss the differences of goal setting between school and clinical site to find common ground. During the discussion, faculty members would be able to convey information about the level of student-acquired learning, and could discuss the gap between what the students learned in school and what they need to learn in the clinical setting.

Securing sufficient time for discussion, faculty members would be better involved in the patient care provided by students in a proactive way. If faculty members are involved in patient care with students, the goals of students and patients become more real.

It would be difficult to solve the problem related to human and time limitations in teaching in a brief space of time. However, faculty members and clinical educators must clarify between themselves the needs and obstructive factors to concrete student achievement; later, they should discuss these with the relevant personnel of the hospitals or school. Faculty members and clinical educators should realize always that the goal of nursing education is excellent care for patients. This will benefit not only patients but also students and the clinical setting directly.

### Limitations

There are two limitations on this research. The first limitation is the small number of participants, and the second limitation is that the data were coded by only one person.

Because of the limited number of participants, especially of faculty members, we might not have achieved theoretical saturation. Additionally, the participants worked in urban areas and the area characteristics may have an effect on the results of the FGIs.

We should conduct further FGIs for more faculty members and clinical educators who are working in various areas to check the consistency of the results, and we should have several coders to improve the validity of analysis.

## Conclusions

The Japanese culture and working conditions affect communication between nursing clinical educators and nursing faculty members. Direct opinion exchange between them is crucial for improving the clinical teaching environment in Japan. Determining the aim and role of each in clinical training and exchanging information between clinical educators and faculty members on student readiness, learning context, and patient information before and during clinical training is important for providing effective clinical practice to nursing students. Additionally, Japanese nursing education institutions should support the improvement of faculty member clinical skills. It would be beneficial to help students learn treatment-related techniques for treating patients and for fostering good relationships between clinical nursing educators and faculty members.

## Competing interests

The authors declare that they have no competing interest.

## Authors’ contributions

MT participated in the design of the study and conducted FGIs, data analysis and the drafting of the manuscript. IK and MT participated in the design of the study and helped to analyze and draft the manuscript. All authors read and approved the final manuscript.

## Pre-publication history

The pre-publication history for this paper can be accessed here:

http://www.biomedcentral.com/1472-6955/11/21/prepub
